# Acromegaly resolution after traumatic brain injury: a case report

**DOI:** 10.1186/1752-1947-8-290

**Published:** 2014-09-02

**Authors:** Alejandro Cob

**Affiliations:** 1 Endocrinología, Clínica Los Yoses, San José, Costa Rica

**Keywords:** Acromegaly, Growth hormone deficiency, Growth hormone–producing pituitary adenoma, Post-traumatic hypopituitarism, Traumatic brain injury

## Abstract

**Introduction:**

Anterior hypopituitarism is a common complication of head trauma, with a prevalence of 30% to 70% among long-term survivors. This is a much higher frequency than previously thought and suggests that most cases of post-traumatic hypopituitarism remain undiagnosed and untreated. Symptoms of hypopituitarism are very unspecific and very similar to those in traumatic brain injury patients in general, which makes hypopituitarism difficult to diagnose. The factors that predict the likelihood of developing hypopituitarism following traumatic brain injury remain poorly understood. The incidence of a specific hormone deficiency is variable, with growth hormone deficiency reported in 18% to 23% of cases.

**Case presentation:**

A 23-year-old Hispanic man with a 2-year history of hypertension and diabetes presented with severe closed-head trauma producing diffuse axonal injury, subarachnoid hemorrhage and a brain concussion. A computed tomography scan showed a pituitary macroadenoma. The patient has clinical features of acromegaly and gigantism without other pituitary hyperfunctional manifestations or mass effect syndrome. A short-term post-traumatic laboratory test showed high levels of insulin like growth factor 1 and growth hormone, which are compatible with a growth hormone–producing pituitary tumor. At the third month post-trauma, the patient’s levels of insulin like growth factor 1 had decreased to low normal levels, with basal low levels of growth hormone. A glucose tolerance test completely suppressed the growth hormone, which confirmed resolution of acromegaly. An insulin tolerance test showed lack of stimulation of growth hormone and cortisol, demonstrating hypopituitarism of both axes.

**Conclusion:**

Even though hypopituitarism is a frequent complication of traumatic brain injury, there are no reports in the literature, to the best of my knowledge, of patients with hyperfunctional pituitary adenomas, such as growth hormone–producing adenoma, that resolved after head trauma. A clear protocol has not yet been established to identify which patients should be screened for hypopituitarism. Predictive factors that might determine the likelihood of developing post-traumatic hypopituitarism have not been clearly established, but there is no evidence of the presence of pituitary adenomas as a risk factor in otherwise healthy patients.

## Introduction

Traumatic brain injury (TBI) is the most common cause of death and disability in young adults living in industrialized countries. Death or hospitalization due to TBI occurs in approximately 180 to 250/100,000 persons per year [1-3]. The population at greatest risk is young adult men under 35 years old. A significant number of survivors demonstrate persistent cognitive, physical and emotional deficits that will prevent functioning at pre-injury levels. Most of the cognitive and neuropsychiatric complications of TBI have been attributed to the post-concussion syndrome, but these morbid features show close resemblance to those seen in patients with anterior hypopituitarism. Hypopituitarism is rarely evident, unless it is associated with diabetes insipidus, because of its relatively subtle clinical features, such as tiredness, weight gain and reduction of muscle bulk. These features might be dismissed as intrinsic to the post-trauma phase. Therefore, it has been postulated that some patients are undiagnosed because of the underlying primary diagnosis [1,2].

A few decades before it was considered a rare complication, and in those cases reported, extensive endocrinological evidence to support the clinical syndrome was often lacking [3-5]. Recently, several studies have shown that post-traumatic hypopituitarism (PTHP) is a common complication, with a prevalence of 30% to 70% among long-term survivors [1,2,5-7]. Pediatric prevalence seems to be similar [6,7]. Autopsy results show pituitary necrosis in up to one-third of patients who have sustained fatal head injuries [1,3].

Reports of the prevalence of anterior hypopituitarism in long-term survivors of TBI vary. In the acute post-traumatic phase, the frequency of gonadotrophin, adrenocorticotrophin hormone, thyrotropin (TSH) and growth hormone (GH) deficiencies and hyperprolactinemia was to be up to 100%, 52%, 44%, 23% and 52%, respectively [1,2]. In recent studies, researchers have shown that anterior hypopituitarisms resolve in some patients and that most of their recoveries occurred by 6 months. Some patients who had normal responses in the acute phase developed late pituitary dysfunction; however, no new abnormalities were detected later than 6 months. Hyperprolactinemia and gonadotrophin deficiency, even though they are the most frequent abnormalities, are likely to resolve completely in most patients. In one study, almost one-half of patients with initial GH deficiency were found to have recovered normal function at 12 months [2]. The majority of patients tested in the studies cited had closed-head trauma, so it is unclear if penetrating head trauma is associated with different rates of hypopituitarism. To the best of my knowledge, there have been no studies to date of hypopituitarism in patients with acromegaly or other hyperfunctional pituitary diseases, so the incidence of hypopituitarism in this population is unknown.

## Case presentation

A 23-year-old Hispanic man was admitted to the emergency room of a general hospital without companions. He was found on the street and brought to the emergency room by ambulance. He presented with impaired consciousness and a 4cm right temporoparietal region scalp wound received upon sustaining a severe head trauma during an assault.

**Figure 1:**
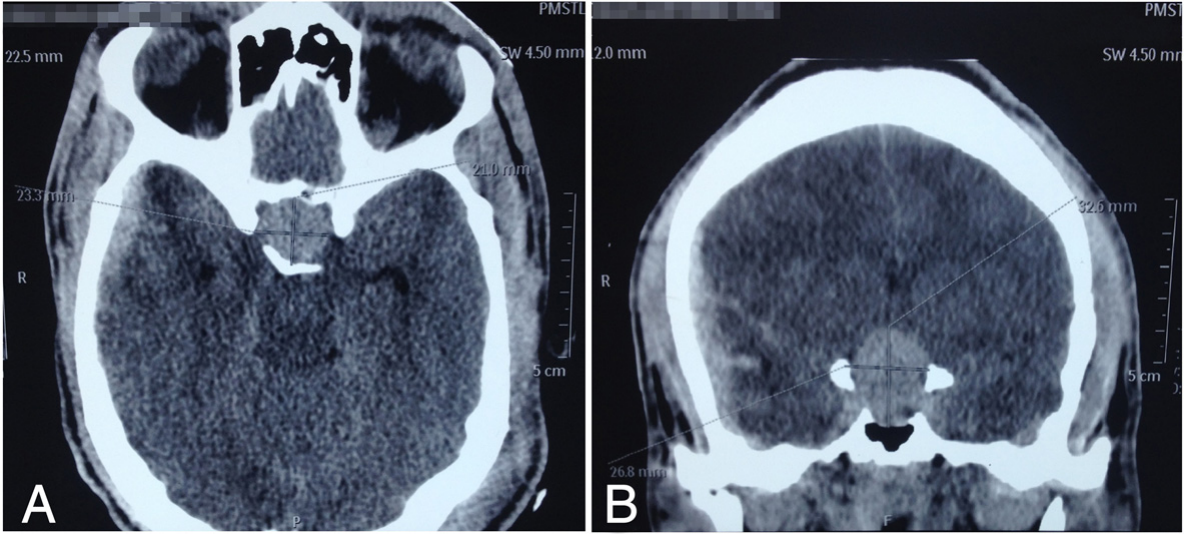
**Initial computed tomography scan after head trauma.** Axial **(A)** and coronal **(B)** computed tomography scans showing the patient’s pituitary macroadenoma, which was 21×26×32mm in size with suprasellar expansion.

At hospital admission, his personal pathological history was unknown. The initial neurological evaluation described him with a Glasgow Outcome Scale (GOS) score of 10 (eye opening = 3, motor response = 6, verbal response = 1). He was also noted to be stuporous and sweaty, with anisocoria, and he responded only to painful stimulation. He required tracheal intubation for airway protection.A head computed tomography (CT) scan revealed diffuse brain edema, right temporal concussion with subarachnoid hemorrhage, a 7mm laminar subdural hemorrhage without mass effect signs or mid-line shift, linear temporal vault fracture, obliterated basal cisterns, concussion of the splenium of the corpus callosum and normal ventricular morphology. Incidentally, a lesion suggestive of a pituitary macroadenoma, 21×26×32mm in size, with suprasellar expansion was observed (Figure 1).

The patient was admitted to the intensive care unit for neurological surveillance and ventilatory support. He required mechanical ventilation for 7 days and antibiotic treatment for a nosocomial pulmonary infection. He developed grade III axonal diffuse injury, but remained hemodynamically stable with mild arterial hypertension.

On the basis of the radiological pituitary finding, an assessment by the endocrinology department was demanded. The patient had clinical coarse facial features suggestive of acromegaly, with prominent supraciliary and suborbital ridges, wide nose, prognathism, increased interdental spaces, increased breadth of feet and hands and features of gigantism. The initial laboratory test revealed insulin-like growth factor 1 (IGF-1) level of 880ng/mL and a GH level of 8.70ng/mL, which are compatible with a GH-producing pituitary tumor. Other hormone laboratory tests were analyzed (Table 1). The patient’s cortisol levels were not measured, because he had been receiving hydrocortisone intravenously since admission to the hospital.

**Table 1:** **Initial hormonal laboratory tests**^**a**^

**Hormone tests**	**Laboratory values**
Free T4	0.764ng/dL
Free T3	3.05pg/mL
TSH	0.212μIU/mL
Prolactin	<0.50ng/mL
GH^b^	8.70ng/mL
IGF-1^c^	880ng/mL
IGF-binding peptide 3	3.51μg/mL
FSH	10.5mIU/mL
LH	4.82mIU/mL

^a^FSH, Follicle-stimulating hormone; GH, Growth hormone; IGF, Insulin-like growth factor; LH, Luteinizing hormone; T3, Triiodothyronine; T4, Thyroxine; TSH, Thyrotropin. ^b^GH normal range = 0.66ng/mL to 5.0ng/mL. ^c^IGF-1 normal range for a 20- 25-year-old = 116ng/mL to 358ng/mL.

After 16 days of hospitalization, the patient’s neurological condition was not stable. An insulin tolerance test was contraindicated. Because of the risk of hypoglycemia in this patient, a thyrotropin-releasing hormone (TRH) stimulation test for TSH was the only stimulatory test performed (Table 2). The rest of the hormonal axes were analyzed after improvement of his critical neurological condition. The TRH stimulation test showed an insufficient TSH response, so a replacement treatment with hydrocortisone and levothyroxine was maintained because of a possible impairment in his pituitary-adrenal/thyroid axes.

**Table 2:** Thyrotropin-releasing hormone stimulation test results

**Time**	**Thyrotropin level**
0 minutes	0.373μIU/mL
20 minutes	1.10μIU/mL
40 minutes	1.06μIU/mL
60 minutes	1.06μIU/mL

In the following three days the patient’s neurological condition improved. He was alert and awake but mildly disoriented, and he was moving his limbs in an uncoordinated manner. He was referred to a rehabilitation center after 23 days of hospitalization.

Eight weeks later after entering the rehabilitation center, the patient was assessed at his first medical appointment as an outpatient in the endocrinology department. He was interrogated for a new and complete medical history. He had been diagnosed with type 2 diabetes mellitus 20 months before sustaining the head trauma, and he was under insulin treatment. He has been the tallest of his four brothers since he was 10 years old. His father’s height is 180cm, and his mother’s is 170cm. He has never worn rings so increase in his fingers width could not be determined, but his shoe size increased during the past year. He indicated that he had excessive sweating prior to the hospital admission. He denied having headaches, visual field defects, diminished libido or sexual dysfunction. He had received insulin treatment during his hospitalization in the rehabilitation center, but it was stopped because of frequent hypoglycemic episodes. The patient’s height was 182cm, his weight was 86.3kg and his blood pressure was 120/80mmHg. He was in good general condition, with no speech abnormalities and no motor sequelae, and with the facial features described above. An examination of his eye fundus and visual field campimetry by confrontation were normal. The laboratory control test showed a decrease from his previous IGF-1 values and a suppression of GH levels (Table 3). His thyroid function tests were normal with levothyroxine therapy.

**Table 3:** **Hormone laboratory control tests**^**a**^

**Hormone tests**	**Laboratory values**
Free T4	1.21ng/dL
TSH	0.553μIU/mL
Prolactin	0.653ng/mL
Cortisol	1.94μg/dL
GH	0.080ng/mL
IGF-1	138.0ng/mL
IGF-binding peptide 3	5.97μg/mL
Testosterone	104.2ng/dL

^a^GH, Growth hormone; IGF, Insulin-like growth factor; T4, Thyroxine; TSH, Thyrotropin.

**Figure 2:**
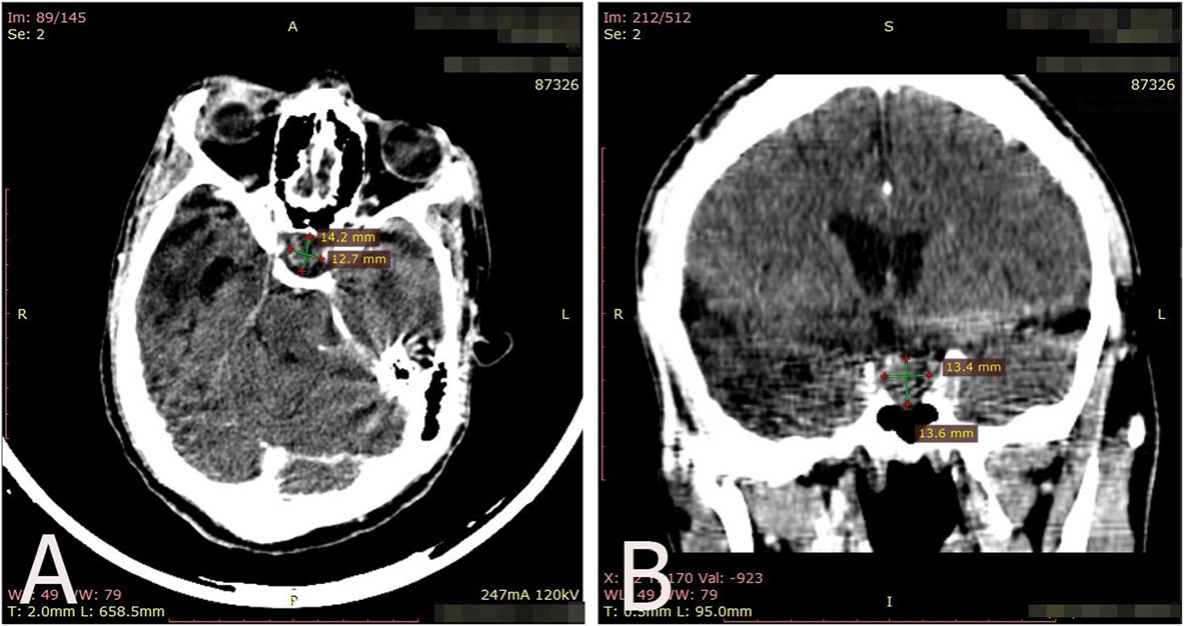
**Control computed tomography scan taken 8 months after head trauma.** Axial **(A)** and coronal **(B)** computed tomography scans show a reduction of the pituitary size with no evidence of a tumor.

Further hormone laboratory tests were performed to analyze the patient’s acromegaly diagnosis and PTHP (Tables 4 and 5). The glucose tolerance test demonstrated a suppressed GH level, which contradicted the initial laboratory and clinical findings of acromegaly. Additionally, the insulin tolerance test showed a lack of stimulation of the GH and cortisol. The control CT scan showed a wide sella turcica with a thin floor wall and a 13×13×14mm homogeneous hypophysis with no specific focalizations (Figure 2).

**Table 4:** **75g glucose tolerance test results**^**a**^

**Time (min)**	**GH**^**a **^**(ng/mL)**	**Glucose (mg/dL)**
0	0.107	111
30	0.11	129
60	0.111	89
90	0.084	99
120	0.09	68

^a^GH, Growth hormone.

**Table 5:** **Insulin tolerance test results**^**a**^

**Time (min)**	**GH**^**a **^**(ng/mL)**	**Cortisol (μg/dL)**	**Glucose (mg/dL)**
0	0.097	4.12	78
30	0.119	3.01	39
60	0.203	4.56	55
90	0.138	4.40	62
120	0.109	2.63	73

^a^GH, Growth hormone.

## Discussion

This patient has clinical features of acromegaly, including prominent supraciliary and suborbital ridges, a wide nose, prognathism, increased interdental spaces and increased breadth of feet and hands, all of which are supported by the CT scan findings of a pituitary macroadenoma and by the initial laboratory test showing high GH and IGF-1 levels. After sustaining head trauma, the patient’s IGF-1 decreased to subnormal levels without any therapeutic intervention. Because GH has a pulsatile secretion pattern during the day, dynamic pituitary testing was performed to confirm the diagnosis. The further glucose tolerance test showed normal suppression of GH. This result confirms acromegaly resolution. The insulin tolerance test demonstrated a lack of stimulation of GH, showing a deficit in this axis. This finding suggests that the patient had hypopituitarism due to head trauma. Also, the pituitary tumor decreased in size after the head trauma. This might be the first reported patient with acromegaly who had resolution of a GH-producing pituitary tumor after head trauma and concomitant development of hypopituitarism.

Even though there was a clear chronological relation between the head trauma and the decrease in GH and IGF-1 levels, there are causes other than head trauma that might trigger pituitary apoplexy. Intrinsic vasculopathy or a rapid increase in size of the intrasellar content leads to increased intrasellar pressure and compression of the pituitary portal blood supply with subsequent tumor shrinkage [8].

Researchers have been trying to identify the factors that might predict the likelihood of developing PTHP, but they still do not fully understand the entirety of the physiopathological mechanisms underlying this condition [1,3,5]. The severity of trauma has been proposed as a risk factor. Most researchers have used the GOS to assess severity of injury, but in some studies no association was found between initial GOS score and hypopituitarism [1,5]. This is probably because the GOS is a variable subjective scale, and responses may be affected by factors others than severity of head injury, such as alcohol consumption.

Recently, researchers have shown some relationship with other factors, such as sex, age, hypoxia, hypotension, polytrauma, transient diabetes insipidus, length of stay in the intensive care unit, cerebrospinal fluid leakage, placement of external ventricular drainage, increased intracranial pressure, duration of tracheal intubation and optic nerve lesions, but a clear association is still lacking [3,5].

Diffuse axonal injury and age have been related to a higher prevalence of hypopituitarism [3,5], and, remarkably, cranial vault fracture has been reported to be associated with a lower prevalence of hypopituitarism. This last finding is difficult to explain. It might be because patients with this injury are often hospitalized even in the absence of severe brain injury. Basal skull fracture has shown a higher incidence of PTHP, and, because of its close proximity to the pituitary, this is not surprising. Similarly, traumatic chiasmal lesions have been considered indirect signs of damage.

Schneider *et al*. [5] reported that there were more patients with PTHP in their optic nerve lesions group, but the difference was not significant, perhaps because of the low absolute rate of this complication. Patients who were in high-speed accidents had a higher incidence of PTHP than patients with accidental hits on the head or penetrating wounds of the brain. Even though hypogonadism was more frequent in patients who had transient diabetes insipidus, polytrauma and hypoxia and in whom external ventricular drains had been placed, it may be a non-specific transient result of critical illness and therefore might represent a physiological down-regulation rather than a pituitary function impairment. Age is a well-known prognostic factor for a worse outcome after TBI. Also, GH secretion and serum testosterone levels decline with age, so it is possible that diagnostic cut-offs for these hormone deficiencies are more likely to be met in patients with initially low levels [5].

Clinical imaging is a useful method of identifying structural brain damage. Investigators are trying to determine if abnormal images of the hypothalamus and pituitary can be used to predict the risk of PTHP. Not all researchers have established an association with CT scan findings [1,6]. Abnormal pituitary clinical imaging results, usually showing vascular injuries, have been reported in >85% of patients with closed-head TBI and PTHP [1,3].

The negative predictive value of severe TBI, abnormal CT scan findings, increased intracranial pressure and intubation for more than 1 day for the development of hypopituitarism is very high, whereas their positive predictive value is low.

## Conclusion

Screening for pituitary function after TBI is an important step toward reducing morbidity and improving outcome [1,5]. Recently published data suggest that PTHP may impair physical rehabilitation and functional outcome after TBI [6]. GH deficiency may also slow recovery because of the associated reduced lean body mass, decreased exercise capacity, impaired cardiac function and reduced bone mineral density, which may be particularly relevant when sex steroid deficiency is also present. In addition, the poor quality of life of patients with GH deficiency may exacerbate any underlying cognitive and neuropsychiatric morbidity [1].

Although most of the available data fail to show an association between PTHP and the severity of trauma, most patients have sustained significant head trauma, defined as those with GCS scores of 3 to 13 or CT scan evidence of brain injury, so they are much more likely to continue to have long-term morbidity. Therefore, this group will benefit most from routine screening to identify and treat PTHP. Patients with milder head injury should be screened if clinically indicated [1]. Early and late abnormalities may be detected, so periodic evaluation in the first year is needed. The problem is that routine testing may not be feasible in every case, owing to the effort and cost of thorough endocrinological testing [6].

The most accepted information to date indicates that diffuse axonal injury, basal skull fractures and age are possible red flags for the need for endocrine testing. Assessment of the GH, gonadal and thyroid axes is not necessary in the acute phase, because there is no evidence that replacement of these hormones improves outcome. The tests are recommended between 3 and 6 months (post–acute phase) and repeated at 1 year [1,6]. The identification and appropriate and timely management of PTHP may significantly aid patient recovery and improve the outcomes of patients with TBI.

This is a unique case report of PTHP in a patient with a GH-producing adenoma and clinical features of acromegaly whose GH hypersecretion resolved 3 months after severe head trauma and whose tumor disappeared, based on CT scans, 8 months later. During his hospitalization, he presented several proposed risk factors for PTHP development, such as diffuse axonal injury, tracheal intubation, a prolonged stay in the critical care unit and a GOS score of 10.

The combination of the resolution of hypersecretion of a functional pituitary adenoma and the tumor disappearance may suggest that the pituitary sustained ischemic damage as a consequence of head trauma. It is necessary to wait for more reports of the association of severe TBI with previous pituitary adenomas to establish whether the clinical course seen in the present case may be an expected or frequent outcome.

## Consent

Written informed consent was obtained from the patient for publication of this case report and any accompanying images. A copy of the written consent is available for review by the Editor-in-Chief of this journal.

## Abbreviations

CT: Computed tomography; GH: Growth hormone; GOS: Glasgow Outcome Scale; IGF-1: Insulin-like growth factor 1; PTHP: Post-traumatic hypopituitarism; TBI: Traumatic brain injury; TSH: Thyrotropin.

## Competing interests

The author declares that he has no competing interests.

## Authors’ contributions

AC analyzed and interpreted the patient data and was the only writer of the manuscript.
